# G protein coupled receptor transcripts in human immune cells and platelets

**DOI:** 10.1038/s41597-024-03880-2

**Published:** 2024-09-27

**Authors:** Arne Hansen, Daniel Martin, Florian Langer, Kathleen Harrison, John Kehrl, Claudia Cicala, Elena Martinelli, Michael J. Brownstein, Eva Mezey

**Affiliations:** 1 Department of Experimental Pharmacology and Toxicology, University Medical Center Hamburg-Eppendorf, 20246 Hamburg, Germany; 2 German Center for Heart Research (DZHK), Partner site Hamburg/Lübeck/Kiel, Hamburg, Germany; 3 Genomics and Computational Biology Core, NIDCR, NIH, Bethesda, MD 20892 USA; 4 Department of Hematology and Oncology, University Medical Center Hamburg-Eppendorf, 20246 Hamburg, Germany; 5 B-Cell Molecular Immunology Section, NIAID, Bethesda, MD 20892 USA; 6 Laboratory of Immunoregulation, NIAID, NIH, Bethesda, MD 20892 USA; 7 Feinberg School of Medicine, Northwestern University, 303 E Superior, Chicago, IL 60611 USA; 8 Azevan Pharmaceuticals, Bethlehem, PA 18015 USA; 9 Adult Stem Cell Section, NIDCR, NIH, Bethesda, MD 20892 USA

**Keywords:** Drug development, Receptor pharmacology

## Abstract

G-protein coupled receptors (GPCRs) are encoded by nonabundant mRNAs, and it is difficult to detect them reliably with the highly parallel methods that are in general use. Because of this, we developed and validated a sensitive, specific, semi-quantitative method for detecting these transcripts. We have used the method to profile GPCR transcripts in white blood cells (WBCs)–B, CD4, CD8, NK, and dendritic cells; monocytes, and macrophage-like monocytes treated with granulocyte-macrophage colony-stimulating factor–as well as platelets. On average, the white cells studied expressed 160 receptor mRNAs (range, 123–206). Platelets made 69. Some, but far from all, of the receptors we found have been detected earlier. We believe our data should stimulate studies of receptor function and contribute to drug development.

## Background & Summary

In 2007 we published a detailed description of a method that could be used to profile GPCR mRNAs in cell and tissue samples^1^. The technique is based on multiplex PCR with panels of specific fluorescent primers. The products that are produced are hybridized to arrays of target DNAs and the slides are then scanned. Two amounts of mRNA are used in the reactions: 10 ng and 100 ng. Products that are detected when 10 ng are employed represent relatively abundant mRNAs. Ones that are only detected when 100 ng are used are rarer.

We have profiled many mRNA samples at this point and believe that the results represent a useful starting point for studies of cell-cell interactions. In addition, they may reveal potential targets for drug development. For example, our profiling data suggested that hematopoietic stem cells (HSCs) make vasopressin receptors, and it appears that activating these increases RBC production and could represent a novel treatment for anaemia^2^.

In this paper, we share our white blood cell and platelet GPCR profiles.

## Methods

All primary white blood cells were obtained from healthy volunteers. All donors provided informed consent that left-over blood donation products can be used for scientific purposes.

### B cells

Human Peripheral Blood Mononuclear Cells (PBMCs) were isolated by Ficoll-Hypaque density gradient centrifugation, incubated in the presence of a CD19 monoclonal antibody (PharMingen) solution for 15 min, and washed in FACS staining buffer–Phosphate Buffered Saline (PBS) without Ca2+/Mg2+ plus 1% BSA. Using a FACStarPlus (Becton Dickinson), the cells in the CD19-positive gate were sorted. Reanalysis of the sorted population by flow cytometry indicated a population purity of greater than 97%.

### CD4 and CD8 T cells and NK cells

PBMCs from single donors were resuspended in PBS with 2% heat-inactivated fetal calf serum and 2 mM EDTA. CD4^+^ T cells, CD8^+^ T cells and NK cells were isolated by negative selection using StemCell Human enrichment kits (StemCell Technologies, Cambridge, MA) as specified by the manufacturer. The cells were shown to be greater than 95% pure using flow cytometry and anti-human CD3, CD4, CD8, CD56, and CD14-20 (Lin-) antibodies.

### Monocytes, macrophages, and immature dendritic cells

CD14+ monocytes from single donors were isolated from PBMCs of unidentified healthy volunteers using immunomagnetic negative selection (Monocyte isolation kit StemCell Technology Vancouver, Canada) as specified by the manufacturer. Isolated monocytes were used as is or cultured to differentiate into macrophages or immature dendritic cells. To differentiate unpolarized macrophages, monocytes were cultured in six-well plates for 6–8 days and fed every 2 days with RPMI 1640 (Bio-Whittaker, Walkersville, Md.) supplemented with 10% heat-inactivated fetal calf serum (HyClone Laboratories, Ogden, Utah), 5% heat-inactivated human serum (Sigma, St. Louis, Mo.), and macrophage colony-stimulating factor (0.1 μg/ml) (Peprotech, Frederick, Md.)^3^. To differentiate immature dendritic cells^4^, monocytes were cultured for 6 days at 1 × 10^6^ cells/ml in 6 well plates in the in the presence of granulocyte-macrophage colony-stimulating factor (GMCSF, 100 ng/ml) and IL4 (50 ng/ml) both from R & D in RPMI 1640, 10% FCS.

### Platelets

Human platelet packs were obtained from 5 healthy donors at the University Medical Center Hamburg-Eppendorf Blood Bank. The platelets were centrifuged twice for 10 minutes at 120 x g, and then washed in modified Tyrode’s buffer (137 mM NaCl, 2.7 mM KCl, 1 mM MgCl_2_, 3.3 mM NaH_2_PO_4_, 5.55 mM glucose and 20 mM HEPES, pH 7.4), pelleted again, and suspended in PBS. The final platelet preparation appeared to be free from contaminating leukocytes based on automated cell counting on a COULTER® Ac·T diff^TM^ Analyzer (Beckman Coulter).

### mRNA preparation

Total RNA was made from the cell samples described above using Ultraspec RNA kits (Biotecx Laboratories Inc., Houston, TX) according to the manufacturer’s instructions. After extraction the RNA was analyzed by spectrometer to determine the concentration and verify a 260/280 ratio of approximately 2.0.

### GPCR transcript profiling

**Fig. 1:**
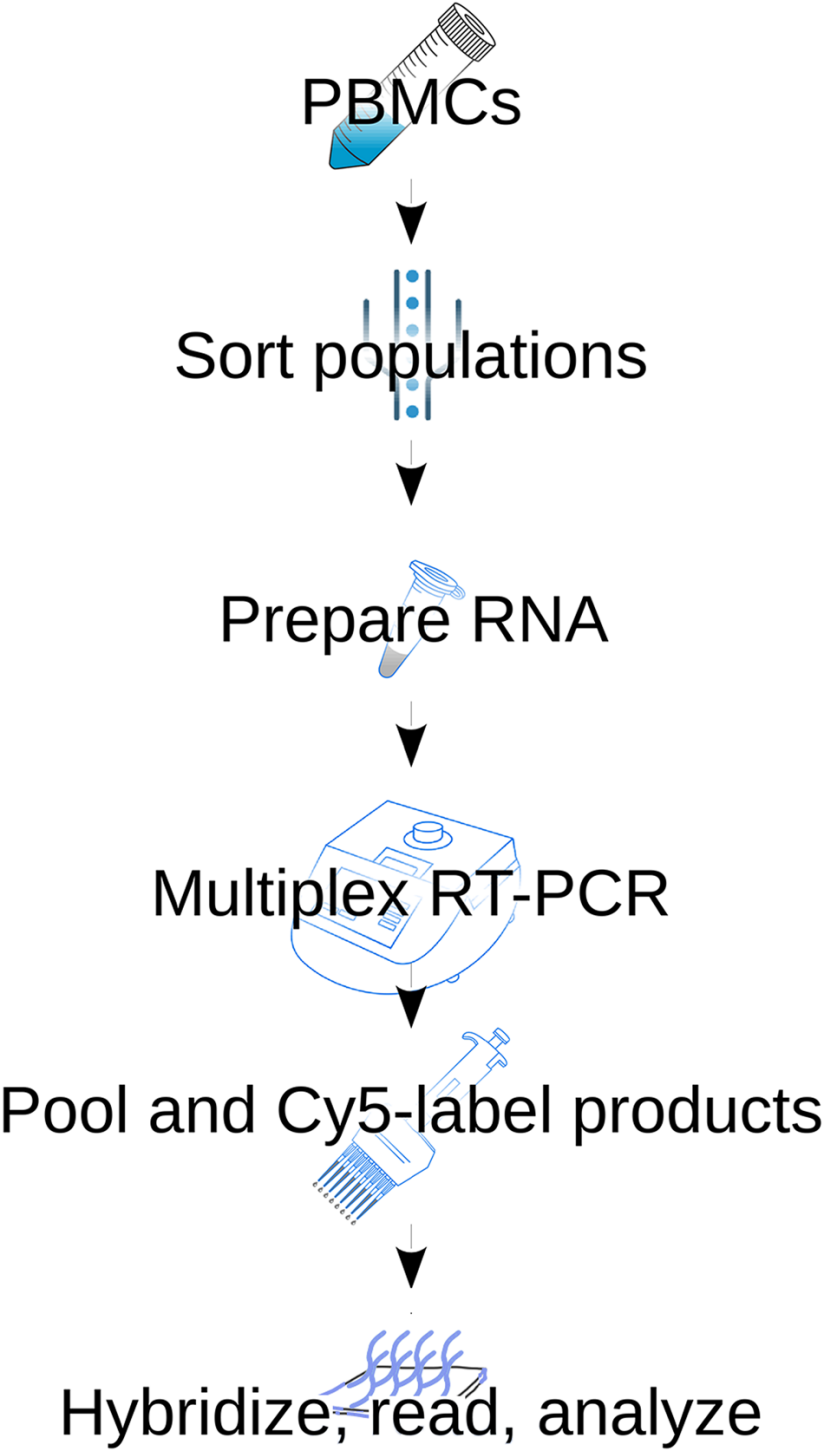
Experimental workflow. PBMCs were collected from healthy donors and sorted into different cell populations. RNA was prepared and its quality and concentration assayed according to standardized techniques. A total of 100 ng per reaction were used as input material to amplify GPCR transcripts in eight separate reactions each containing 50 GPCR specific primer pairs and aminoallyl-dUTP. Separate reactions with 10 ng of input material were used to determine those receptors expressed at higher levels. PCR reactions were pooled and Cy5 labelled on the amino-modified nucleotides. Labelled PCR products were hybridized to custom GPCR arrays that contained plus and minus sense oligonucleotides. Arrays were processed and scanned, and their signal extracted analyzed.

Optimization of assay parameters and detection of GPCR mRNAs was performed as described in Hansen, *et al*.^1^ (Fig. 1). In brief, RNA was purified from the platelets and cells and contaminating DNA was removed with DNase. Eight sets of 50 primer pairs were used to amplify the GPCR mRNAs present 10 ng and 100 ng of each RNA sample. These reactions were run in triplicate. Aminoallyl-modified dUTP was incorporated into the amplicons. After the PCR reactions were performed, the products of 4 reactions (200 potential products) were pooled. Thus, two pools (x3) were created for each of the two concentrations of RNA studied (10 ng and 100 ng), then the products were labelled by coupling the NHS ester of Cy5 to the amino-modified bases that had been incorporated into the amplicons. The final labelled products were hybridized to glass-slide arrays printed with both plus and minus sense oligonucleotides so that the products of balanced or unbalanced reactions generate signals. Finally, the arrays were read, and the signals were analyzed^1^. Receptor mRNAs are reported to be present below if all three replicates were positive. Nearly all the RNAs detected in 10 ng of template were also found in 100 ng. Fewer transcripts were detected in 10 ng of template than in 100 ng reflecting the fact that only abundant receptor RNAs are detectable when the smaller amounts of template are used.

### Analyzes

A GPCR expression call was made if amplification was detected in 3 out of 3 independent technical replicates (e.g. separate PCR amplification, labeling and hybridization). Because we used two amounts of input material per reaction, we defined GPCRs as being expressed if they were amplified in 100 ng of input material and expressed at high levels if they were also amplified in 10 ng of input material. A table of GPCR expression calls was assembled in Excel with the results of the triplicate amplifications and subsequent analyzes were performed using pivot tables. A harmonized GPCR symbol space was produced by iterative manual curation of ours and Groot-Komerlink *et al*.^5^ datasets using the web-based ToppGene^6^ tool and the HGNC database (genenames.org)^7^. GPCR symbols are current as of March 2024.

## Data Records

We assayed 382 GPCR mRNAs. Their names (based on HUGO) are listed in the G protein coupled receptor transcripts in human immune cells and platelets database^8^, which includes all the assay data.

As noted, the PCR reactions were done in triplicate with two amounts of template-10 ng and 100 ng and all the receptors detected in 10 ng of template were also detected in 100 ng. Those that were only detected in 100 ng represent less abundant mRNAs than those that are also detected in 10 ng of template.

In the various white blood cells (WBCs) we studied, we detected a total of 265 GPCR transcripts. One hundred and sixty-two of these appeared to be relatively abundant (detected in 10 ng of RNA) in at least one cell type, and 103 were relatively rare (detected exclusively in 100 ng of RNA).

**Fig. 2:**
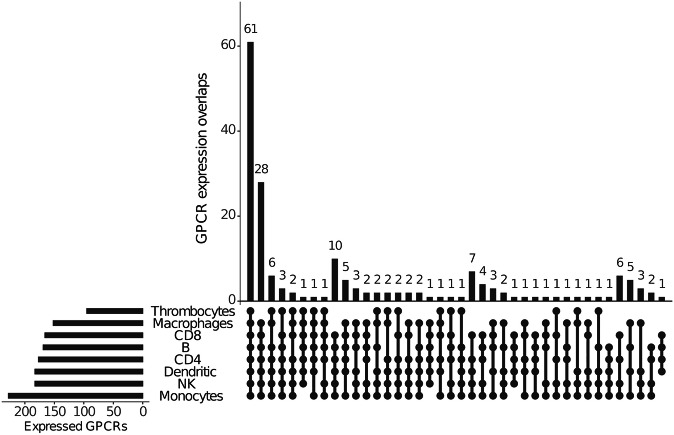
Analysis of individual GPCR expression overlaps. Bars indicate the number of GPCR shared by the cell types indicated by connected black dots in the cell matrix. The total amount of GPCRs expressed by each cell type is indicated by the horizontal bars in the lower left quadrant.

Sixty-one GPCR mRNAs were detected in *all* the 100 ng samples (Table 1a and Fig. 2). If platelets are excluded, the number increases to 89 (Table 1b). TRPA1 was expressed at high levels in all the RNAs except the one from platelets. One hundred and two of the RNAs were detected in 100 ng of 7 of the 8 samples (Table 2a). Only 11 of these were detected in 10 ng (Table 2b). Some relatively abundant receptors appear to be unique to each sample studied (Table 3).

**Table 1:** GPCR mRNAs that are expressed by all cell types assayed.

**a. GPCR mRNAs detected in all WBCs and platelets**.
*ADGRE4P, C5AR2, CCRL2, CXCR2, CXCR3, CXCR4, CYSLTR1, F2R, F2RL1, F2RL2, FFAR3, GPR108, GPR132, GPR135, GPR146, GPR155, GPR174, GPR183, GPR19, GPR20, GPR21, GPR27, GPR37, GPR55, GPR68, GPR75, GPR82, GPR83, GPR84, GPR89, GRM2, GRM4, HCAR2, HCAR3, HTR2B, LGR4, LPAR2, LPAR3, LPAR5, LTB4R, LTB4R2, MC1R, NPBWR1, NTSR1, OPN3, OXER1, P2RY11, P2RY8, PTAFR, PTGIR, RRH, RXFP3, S1PR1, S1PR2, S1PR4, S1PR5, SMO, TBXA2R, TM7SF3, VN1R1, XCR1*
**b. GPCR mRNAs detected in all WBCs**.
*ACKR3, ADGRA2, ADGRB1, ADGRD1, ADGRE4P, ADGRE5, ADRA2B, ADRB1, C5AR2, CCRL2, CELSR2, CXCR2, CXCR3, CXCR4, CYSLTR1, F2R, F2RL1, F2RL2, FFAR3, FZD1, FZD2, FZD3, FZD6, GIPR, GPR108, GPR132, GPR135, GPR142, GPR146, GPR151, GPR155, GPR157, GPR161, GPR174, GPR183, GPR19, GPR20, GPR21, GPR25, GPR27, GPR3, GPR35, GPR37, GPR55, GPR65, GPR68, GPR75, GPR82, GPR83, GPR84, GPR89, GRM2, GRM4, HCAR2, HCAR3, HTR2B, LANCL2, LGR4, LPAR2, LPAR3, LPAR5, LPAR6, LTB4R, LTB4R2, MC1R, NPBWR1, NTSR1, OPN3, OPRL1, OXER1, P2RY11, P2RY4, P2RY8, PTAFR, PTGIR, RRH, RXFP3, S1PR1, S1PR2, S1PR4, S1PR5, SLC52A1, SMO, TAS2R14, TAS2R5, TBXA2R, TM7SF3, VN1R1, XCR1*

**Table 2:** GPCR mRNAs that are expressed by most cell types assayed.

**a. GPCR mRNAs found in at least 7 out of 8 cell types**.
*ACKR2, ACKR3, ADGRA2, ADGRB1, ADGRD1, ADGRE1, ADGRE4P, ADGRE5, ADORA2B, ADRA2B, ADRB1, C3AR1, C5AR2, CCRL2, CELSR2, CXCR2, CXCR3, CXCR4, CXCR5, CYSLTR1, F2R, F2RL1, F2RL2, FFAR1, FFAR2, FFAR3, FZD1, FZD2, FZD3, FZD6, GIPR, GPR108, GPR132, GPR135, GPR142, GPR146, GPR151, GPR155, GPR157, GPR161, GPR174, GPR183, GPR19, GPR20, GPR21, GPR25, GPR27, GPR3, GPR35, GPR37, GPR55, GPR65, GPR68, GPR75, GPR82, GPR83, GPR84, GPR89, GRM2, GRM4, HCAR2, HCAR3, HTR2B, LANCL2, LGR4, LPAR1, LPAR2, LPAR3, LPAR5, LPAR6, LTB4R, LTB4R2, MC1R, MRGPRE, NPBWR1, NTSR1, OPN3, OPRL1, OXER1, P2RY11, P2RY12, P2RY4, P2RY8, PTAFR, PTGER3, PTGIR, RRH, RXFP3, S1PR1, S1PR2, S1PR4, S1PR5, SLC52A1, SMO, TACR2, TAS2R14, TAS2R5, TBXA2R, TM7SF3, TPRA1, VN1R1, XCR1*
**b. GPCR mRNAs present at high levels in at least 7 out of 8 cell types**.
*FFAR3, GPR108, GRM2, GRM4, LPAR2, LPAR5, P2RY8, S1PR4, TBXA2R, TPRA1, VN1R1*

**Table 3:** GPCRs that are uniquely expressed by specific cells, bold ones are at high levels.

B	**ADGRF1, GPR18, NPFFR1**, GCGR, MRGPRX4
CD4	**DRD2, FZD6, OPRD1, SLC52A1, SSTR3**, BRS3, CXCR6, MRGPRD
CD8	**CELSR2, LPAR3, TAAR8**
Dendritic Cells	**CMKLR1, FPR3, GPR153, GPR161, HCAR1, HRH1, P2RY14, P2RY6**, HCRTR2, HTR1D, MAS1L, RHO
Macrophages	ADRA2C, CRHR2, GPR156
Monocytes	**ADGRD1, ADGRE1, ADGRE2, ADGRE3, ADORA2B, ADRB1, ADRB2, C3AR1, C5AR1, CCR2, CCR3, CCRL2, CHRM4, CNR2, CX3CR1, F2RL1, FPR2, GPBAR1, GPR142, GPR34, GPR89, GPRC5B, GRM3, LANCL1, LANCL2, MRGPRE, NTSR1, P2ry13, PTGDR2, SSTR5**, ADGRL3, APLNR, FZD5, GPR141, GPR160, GRM6, HTR1E, HTR1F, HTR7, MC5R, MRGPRX3, MTNR1A, OXGR1, PROKR2, PTGFR
NK	**ADGRB2, ADGRG5, ADGRG7, CCR9, GHSR, GPR52, MC3R, P2RY4**
Thrombocytes	**OXTR, P2RY12**

## Technical Validation

Technical validation of the assay method we used can be found in the paper by Hansen *et al*.^1^. The false positive rate appears to be 1–3%.

**Fig. 3:**
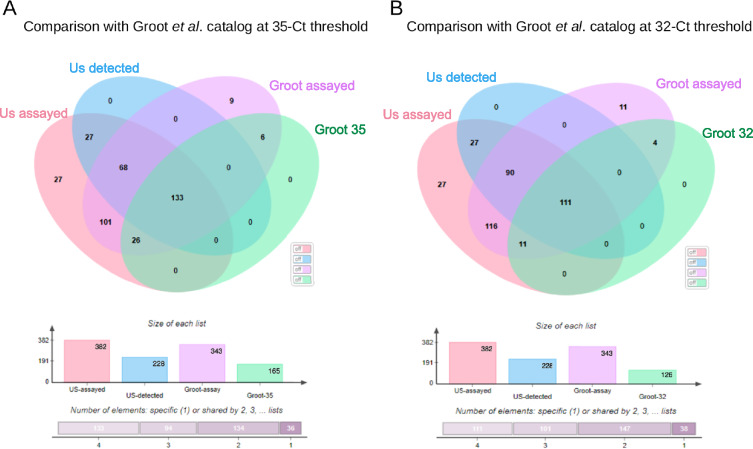
GPCR expression detection method comparison. (**A**) Venn analysis was used to compare the sensitivity of our method compared to the Groot *et al*. study at a Ct threshold of 35 cycles. Us-assayed means the GPCRs assayed in our study. Us-detected, the GPCRs detected in our 100 ng input assay. Groot-assayed, the total amount of GPCRs assayed by Groot-Kormelink *et al*. Groot-35 the GPCRs detected by TaqMan QPCR assays at a 35-cycle threshold in that study. (**B**) Venn analysis was used to compare the sensitivity of our methods compared to the Groot *et al*. study at a Ct threshold of 32 cycles. Us-assayed, the GPCRs assayed in our study. Us-detected, the GPCRs detected in our 100 ng input assay. Groot-assayed, the total amount of GPCRs assayed by Groot-Kormelink *et al*. Groot-32 the GPCRs detected by TaqMan QPCR assays at a 32-cycle threshold in that study.

We looked for studies like ours in the literature, but only two have been published that might help validate our results. One of those papers cultured the monocytes before assaying thus we compared our results with Groot-Kormelink *et al*.^5^ who used q-PCR to assay 365 individual GPCR transcripts in monocytes isolated from 5 subjects (Fig. 3). Recall that we studied 382 receptors. Groot-Kormelink *et al*.^5^ looked at 25 mRNAs that we omitted, but only 11 of these are annotated as GPCR transcripts (Table 4).

**Table 4:** Extra genes in *Groot-Kormelink et al*. monocyte dataset. Bona-fide GPCRs in **bold fonts**.

SYMBOL	Description	EntrezID
LONP2	LONP2 (lon peptidase 2, peroxisomal)	83752
SENP3	SENP3 (SUMO specific peptidase 3)	26168
HDAC3	HDAC3 (histone deacetylase 3)	8841
**GPR137**	GPR137 (G protein-coupled receptor 137)	56834
PTPN22	PTPN22 (protein tyrosine phosphatase non-receptor type 22)	26191
**OR2A4**	OR2A4 (olfactory receptor family 2 subfamily A member 4)	79541
MATK	MATK (megakaryocyte-associated tyrosine kinase)	4145
TRBV5-4	TRBV5-4 (T cell receptor beta variable 5-4)	28611
PHGDH	PHGDH (phosphoglycerate dehydrogenase)	26227
NPY6R	NPY6R (neuropeptide Y receptor Y6 (pseudogene))	4888
**OR2C3**	OR2C3 (olfactory receptor family 2 subfamily C member 3)	81472
GCNT2	GCNT2 (glucosaminyl (N-acetyl) transferase 2 (I blood group))	2651
**OR7C2**	OR7C2 (olfactory receptor family 7 subfamily C member 2)	26658
**VN1R5**	VN1R5 (vomeronasal 1 receptor 5 (gene/pseudogene))	317705
**ADGRF4**	ADGRF4 (adhesion G protein-coupled receptor F4)	221393
**GPR26**	GPR26 (G protein-coupled receptor 26)	2849
VN1R10P	VN1R10P (vomeronasal 1 receptor 10 pseudogene)	387316
**VN1R2**	VN1R2 (vomeronasal 1 receptor 2)	317701
**GNRHR2**	GNRHR2 (gonadotropin releasing hormone receptor 2 (pseudogene))	114814
**TAS1R1**	TAS1R1 (taste 1 receptor member 1)	80835
**OR7E5P**	OR7E5P (olfactory receptor family 7 subfamily E member 5 pseudogene)	219445
PCDH15	PCDH15 (protocadherin related 15)	65217
SLC26A7	SLC26A7 (solute carrier family 26 member 7)	

We assayed 42 receptor mRNAs that they neglected. Using a threshold of 32 cycles as the cutoff for “expression”, they found 126 GPCR transcripts in monocytes and we found 228 transcripts in 100 ng of RNA. We both detected 111 of the receptor RNAs. They found 4 that we omitted and 11 that we assayed but did not detect. We assayed 54 receptor RNAs that they did not look for and detected half of them. We failed to detect 11 of the receptors that they found, and they did not see 9 of the receptors that we saw. If we increase their threshold for expression to 35 cycles, they detected 165 GPCR mRNAs, 133 of which we also saw. We did not assay 6 of the receptors they found and did not detect 26 of them. Perhaps this should have been detected. The comparison between both assays is summarized in Fig. 3. The assays that both groups used were difficult and time consuming. We looked at single cell samples. Had both of us studied more cells, we might have seen additional overlap that may have been more extensive. The non-abundant receptor mRNAs that we did detect may or may not have been translated. This would have to be determined for one receptor at a time. On the other hand, their presence may hint that they have significant roles. We did not study cells harvested from non-normal patients. This might have revealed additional transcripts or a shift in the abundance of those we detected. The full comparison between the studies and results are summarized in the G protein coupled receptor transcripts in human immune cells and platelets database^8^ in Excel file format.

## Data Availability

No custom code was used in data analysis. The data are shown in excel sheets in pivot tables and manual curation.
